# Megakaryoblasts in pleural fluid in neonate with transient leukaemia of Down's syndrome

**DOI:** 10.1002/jha2.692

**Published:** 2023-04-21

**Authors:** Karolina Chmielowiec, Gary Beattie, Kathryn Clarke, Ellen McCloy, Bethany Mitchell, Christine Macartney

**Affiliations:** ^1^ Department of Haematology Belfast City Hospital Belfast Northern Ireland; ^2^ Department of Paediatric Haematology Royal Belfast Hospital for Sick Children Belfast Northern Ireland

**Keywords:** childhood leukaemia, leukaemia morphology, megakaryocytes

1

Twin baby boy born at 35+2 weeks gestation with a birth weight of 2070 grams via emergency cesarean section secondary to abnormal CTG and foetal distress. Full blood count obtained at birth showed haemoglobin 159 g/L, total white cell count 99.54 × 10^9^/L platelet count 779 × 10^9^/L. Manual differential showed an absolute neutrophil count of 2.01 × 10^9^/L absolute lymphocyte count of 45.27 × 10^9^/L and blast count of 50.3 × 10^9^/L (50%). Initial peripheral blood smear morphology review revealed a thrombocytosis, platelet anisocytosis and micromegakaryocytes. It also demonstrated neutropenia, myeloid left shift, large blasts with a high N/C ratio and nucleoli. Additionally, numerous nucleated red blood cells were seen with marked red cell dysplasia including binucleate forms and abnormal haemoglobinisation. There was no prenatal diagnosis of Down's syndrome (DS). The above blood results along with the baby's clinical signs raised suspicion of DS and subsequently transient leukaemia of DS. This baby was then transferred to the regional neonatal intensive care unit on day 2 of life.

On admission, he was noted to have bilateral pleural effusion, synthetic liver dysfunction with elevated creatinine and ammonia levels as well as ascites. He was intubated, and ventilated and required inotropic support. Due to adverse features of elevated white cell count, hepatosplenomegaly, pleural effusion as well as ascites [1] and following the multidisciplinary team (MDT) discussion and parental consent, the patient was commenced on low dose cytarabine for 7 days. He tolerated that well, however, did experience mild cytopenias and pyrexic episodes, followed by recovery during the following two weeks with a reduction in pleural effusions, hepatosplenomegaly and reduction in oxygen requirement which enabled successful extubation and commencement of continuous positive airway pressure.

Unfortunately, on day 19 of life he was noted to have slowly increasing oxygen requirements with re‐accumulation of pleural effusions and increasing hepatosplenomegaly as well as increasing blast count in peripheral blood. He proceeded to require intubation and ventilation again as well as placement of a chest drain to remove pleural fluid to improve respiratory compliance. A review of pleural fluid morphology had shown frequent ‘blast‐like’ cells; these were medium‐sized with a high N/C ratio with finely condensed chromatin nucleoli and budded basophilic cytoplasm thought to most likely represent megakaryoblasts [2]. As a result of the deterioration, a decision by MDT was made to give another cycle of low‐dose cytarabine on days 1 through 5. Following the second cycle of cytarabine, the patient again experienced mild cytopenias and pyrexic episodes, was treated with antibiotics and supportive management and transfusions of packed red cells. He had complete count recovery and no blast population identified morphologically on peripheral blood film on day 26 of the second cycle of cytarabine. His *GATA1* mutation was subsequently found to be positive. He continued to improve and was discharged home on day 67 of life Figures 1, 2, 3.

**FIGURE 1:**
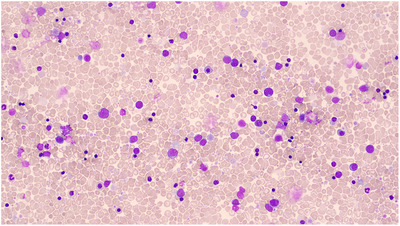
Peripheral blood film on day 1 of life showing thrombocytosis, platelet anisocytosis, micromegakaryocytes, neutropenia, myeloid left shift, large myeloid blasts with high N/C ratio and nucleoli, nucleated red blood cells and red cell dysplasia including binucleate forms and abnormal haemoglobinisation.

**FIGURE 2:**
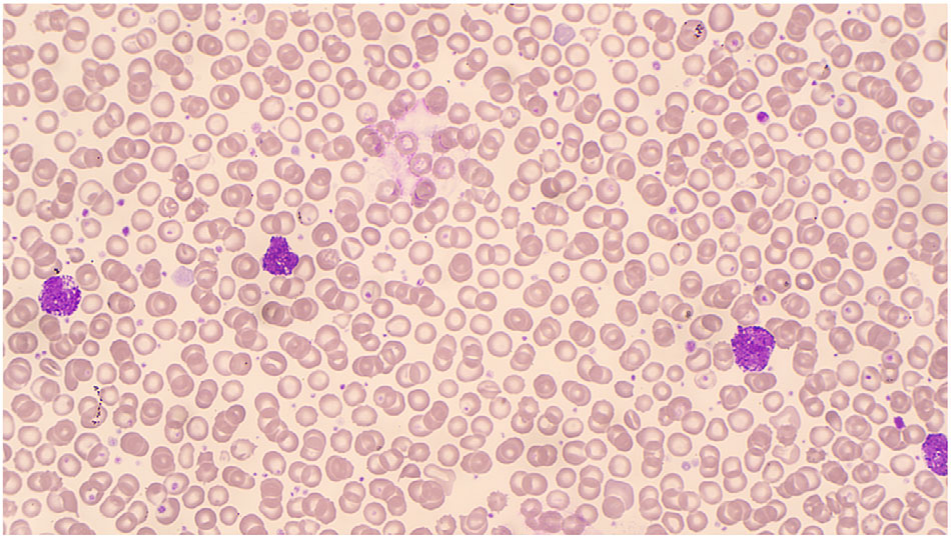
Peripheral blood film on day 21 of life showing a population of medium‐sized blasts, as well as thrombocytosis, platelet anisocytosis and mild polychromasia.

**FIGURE 3:**
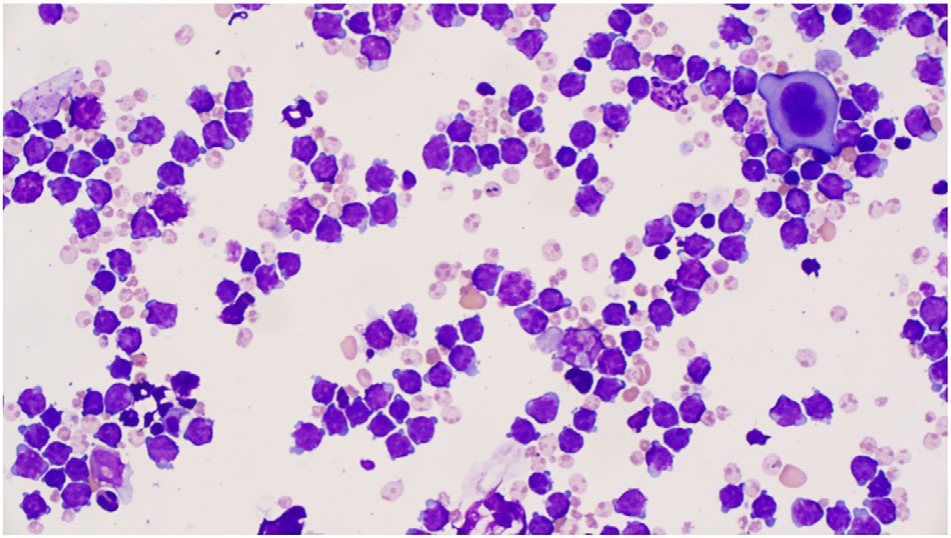
Wright‐Giemsa stain of pleural fluid on day 19 of life showing megakaryoblasts and one maturing megakaryocyte.

## AUTHOR CONTRIBUTIONS

**Karolina Chmielowiec**—wrote the manuscript, **Gary Beattie**—manuscript review, obtained image, obtained and analysed flow cytometry data, **Kathryn Clarke**—manuscript review, obtained and analysed flow cytometry, **Ellen McCloy**—manuscript review, morphology review, **Bethany Mitchell**—manuscript review, morphology review, **Christine Macartney**—manuscript review, supervising consultant. All authors discussed the results and commented on the manuscript.

## CONFLICT OF INTEREST STATEMENT

The authors declare no conflict of interest.

## FUNDING INFORMATION

No funding was required for this submission.

## ETHICS STATEMENT

Not applicable.

## PATIENT CONSENT STATEMENT

Not Applicable.

## CLINICAL TRIAL REGISTRATION

Not Applicable All authors have contributed to the acquisition, analysis and interpretation of data as well as either drafting the paper or revising it critically. All authors approved the submitted and final versions.

## Data Availability

For original data, please contact karolina.chmielowiec@belfasttrust.hscni.net
